# Sensitivity hasn’t got a Heterogeneity Problem - a Reply to Melchior

**DOI:** 10.1007/s11406-016-9782-z

**Published:** 2016-11-14

**Authors:** Kevin Wallbridge

**Affiliations:** Department of Philosophy, University of Edinburgh, 3 Charles st, Edinburgh, EH8 9AD UK

**Keywords:** Sensitivity, Reflective knowledge, Higher-order knowledge, Bootstrapping, Easy knowledge

## Abstract

In a recent paper, Melchior pursues a novel argumentative strategy against the sensitivity condition. His claim is that sensitivity suffers from a ‘heterogeneity problem:’ although some higher-order beliefs are knowable, other, very similar, higher-order beliefs are insensitive and so not knowable. Similarly, the conclusions of some bootstrapping arguments are insensitive, but others are not (and since one motivation for endorsing the sensitivity condition was to provide an explanation of what goes wrong in bootstrapping arguments, this motivation is undermined). In reply, I show that sensitivity does not treat different higher-order beliefs differently in the way that Melchior states and that while genuine bootstrapping arguments have insensitive conclusions, the cases that Melchior describes as sensitive ‘bootstrapping’ arguments don’t deserve the name, since they are a perfectly good way of getting to know their conclusions. In sum, sensitivity doesn’t have a heterogeneity problem.

## Introduction

In a recent paper,^1^ Melchior pursues a novel argumentative strategy against the claim that sensitivity is necessary for knowledge. His claim is that sensitivity suffers from what he calls ‘the heterogeneity problem.’ In a nutshell, the problem is that according to the sensitivity condition although *some* higher-order beliefs are knowable, *other* very similar higher-order beliefs are insensitive and so not knowable, and this is problematic for sensitivity because it seems that the similar higher-order beliefs in question should be similarly knowable. Following on from this result, he argues that the same goes for the conclusions of bootstrapping and Moorean arguments: the sensitivity condition is met in some cases but not others. But one motivation for endorsing the sensitivity condition was to provide an explanation of what goes wrong in bootstrapping arguments, and this won’t work if some bootstrapping arguments have sensitive conclusions.

In reply, I will argue that sensitivity does not treat different higher-order beliefs differently in the way that Melchior states. (As it happens, it does treat *some* higher-order beliefs differently, but only those that intuitively do strike us as epistemically different, because they include the denial of skeptical hypotheses.)

Regarding Bootstrapping and Mooreanism, the case is similar. The genuinely troubling cases have insensitive conclusions, but the cases that Melchior describes as sensitive ‘bootstrapping’ arguments don’t deserve the name, since they are a perfectly good way of getting to know their conclusions. In sum, sensitivity *doesn’t* have a heterogeneity problem. It treats epistemically similar cases the same, as it should.

## Sensitivity

The sensitivity condition is the claim that sensitivity is a necessary requirement for knowledge, where S’s belief that p, formed via method M, is sensitive just in case:

If p were false and S were to use M to arrive at a belief whether (or not) p, then S wouldn’t believe, via M, that p.^2^

(Take careful note of the reference to the method of belief formation - this will be important later.) This counterfactual can then be evaluated in line with the standard Lewis/Stalnaker semantics, i.e. it holds when the consequent is true at the nearest possible worlds in which the antecedent is true.

## Higher-Order Belief

Following others in this debate,^3^ Melchior distinguishes between two important forms of higher-order belief (which may or may not be a good match for what we mean when we say things like ‘My belief that p is true’ or ‘My belief that p is not false’). Those of the form (Bp & p) and those of the form ~ (Bp & ~ p).^4^

He argues that beliefs of the first form (which are conjunctive, so he calls *c*) are sensitive, since so long as your belief that p is sensitive, in the nearest possible world where (Bp & p) is false, you do not believe that p and, therefore, do not believe (Bp & p).^5^

However, there remain beliefs of the latter form (whose content is logically equivalent to a disjunction, so he calls *d*), and he argues (following Vogel)^6^ that beliefs of this form are *in*sensitive. This is because in the nearest worlds in which ~ (Bp & ~ p) is false, since one still believes that p, if one uses the same method of inference as in the actual world to assess whether ~ (Bp & ~ p) then one will still come to believe that ~ (Bp & ~ p).

Melchior claims that this heterogeneity is a problem for sensitivity, since the beliefs (Bp & p) and ~ (Bp & ~ p) are “closely related. Moreover, some of the sensitively believed propositions are stronger than their insensitively believed counterparts. This outcome is a mess.”^7^ Happily, it turns out to be a mess which can easily be tidied up.

To do so, it will be helpful to introduce a concrete case (I’ve chosen one familiar from the literature).

### New Shoes

You see your long-time friend Omar, who is a perfectly decent and straightforward sort of person. Noticing his shiny white footwear, you say, “Nice shoes, Omar, are they new?” Omar replies, “Yes, I bought them yesterday.” You know that Omar has new shoes, and that you believe that Omar has new shoes. You also know, if you think about it, that you don’t falsely believe that Omar has new shoes. (Vogel 2000, 2007, 2012.)

Let p be the proposition that Omar has new shoes. Contrary to Melchior’s claim that higher-order knowledge of the form ~ (Bp & ~ p) is insensitive (i.e. the currently orthodox view, also espoused by Vogel, Sosa, Huemer, Becker, and Salerno), I will show that in this case your belief that ~ (Bp & ~ p) actually *is* sensitive.^8^

While it is true that if it were the case that (Bp & ~ p), you would still falsely believe ~ (Bp & ~ p), importantly, you would not believe it on the same *basis* as you *actually* believe it. Given that Omar is “a perfectly decent and straightforward sort of fellow” it would not easily have been the case that Omar lied to you or was otherwise mistaken about his shoes (if that were the case then your belief that p would not be sensitive). So if you had believed *falsely* that Omar has new shoes then this would have been on some other, less reliable, basis; perhaps that you heard it from some unreliable testifier or perhaps you saw Omar at a distance and mistook his ordinary shoes for new ones. In such a case your belief that you do not falsely believe that Omar has new shoes, i.e. ~(Bp & ~ p), would not be formed on the same basis as it is in the actual world, since in the actual world you form that belief at least partly on the basis that Omar has told you that he has new shoes.^9^

With that in mind, let’s consider whether your belief that ~ (Bp & ~ p) is sensitive. Think about the nearest worlds where this is false, i.e. the nearest (Bp & ~ p) worlds. As we have just seen, those are worlds in which Omar doesn’t tell you that p (but you falsely believe p on the basis of some other less reliable kind of evidence). In these worlds, if you were to use the same method as you did in the actual world to judge whether ~ (Bp & ~ p), would you thereby end up believing that ~ (Bp & ~ p)?

The problem with understanding how to answer this question is that the method that you actually used seemingly involved basing your higher-order belief (partly) on Omar’s telling you that he has new shoes, but of course, in the worlds now under consideration Omar has told you no such thing. Therefore, in such a world one cannot form a belief on the basis of exactly the same kind of evidence as one does in the actual world.

What is required is a nuanced understanding of methods, according to which the same method can be applied in cases with different evidence. In the case at hand, the relevant method would not be *base your belief on Omar’s telling you that he has new shoes* (which isn’t available in all of the worlds we want to consider), but something like *ask Omar about his shoes* or perhaps *base your belief on what Omar tells you about his shoes* (which *is* available in all of the worlds which we want to consider). You can still count as applying this method even if Omar tells you that he doesn’t have new shoes, or if he doesn’t tell you anything at all about his shoes and so even if you don’t form a belief either way on this basis.^10^

Once we have spelt out the relevant method of belief formation, we can see that your belief that ~ (Bp & ~ p) *does* count as sensitive in the *New Shoes* case. If it were false that ~ (Bp & ~ p) and you were to apply this kind of method, then you would not believe (via this method) that ~ (Bp & ~ p). Since Omar is “a perfectly decent and straightforward sort of fellow” he would either have said nothing at all, or else said that his shoes *weren’t* new, and either way you would not form the belief that ~ (Bp & ~ p) via the method *base your belief on what Omar tells you about whether his shoes are new* (even if there also happens to be some other method by which you do so believe).

## Genuinely Insensitive Beliefs

I have shown, contra Melchior, that higher-order beliefs of the form ~ (Bp & ~ p) can be sensitive (along with those of the form (Bp & p)). But there *are some* higher-order beliefs which are insensitive. This has nothing to do with whether they are conjunctive or disjunctive though as Melchior claims and instead has everything to do with their content. The trick is in forcing the sensitivity condition to look at worlds where you believe falsely *while still believing on the basis of the very same evidence that you actually do*:

~(Bp *[on the very same basis as you actually do believe that p]* & ~ p).

This belief is such that if it were false then you would still believe it *on the very same basis* (e.g. on the basis that Omar told you that he has new shoes). As applied to the *New Shoes* situation, the relevant belief would be ~ (Bp *[on the basis that Omar told you that p]* & ~ p).

*That* belief *is* insensitive. However, to be a problem for sensitivity, it also has to be the case that you *know* the proposition in question. But there is no reason to think that you *do* know this. Translated out of the formalism, this would be to say that you know that it is not the case that Omar does not have new shoes but that he has *told you* that he *has*, leading you to falsely believe that he has. In other words, you know that it is not the case that Omar is lying to you, or has forgotten what shoes he is wearing, or misspoke or something. This is in essence to know the denial of a (local) skeptical hypothesis.

But this is not the sort of thing that you can know, at least not in the kind of way at issue, i.e. just by reflection on your belief that Omar has new shoes, and your belief that you believe that Omar has new shoes. *That* is not enough to know that Omar is not lying to you or that he did not misspeak. (Perhaps you *do* know those things already because you know independently that Omar always tells the truth and never misspeaks, or you could *come to know* that Omar did not lie or misspeak if you checked in some independent way that Omar genuinely does have new shoes, say if you checked his bank statement or the CCTV footage of the shoe shop - in these cases your belief would be sensitive.)

## Bootstrapping and Moorean Arguments

After making his initial arguments regarding the heterogeneous way that sensitivity treats beliefs of the forms ~ (Bp & ~ p) and (Bp & p) Melchior goes on to argue that this undermines sensitivity theorists’ response to cases of bootstrapping and Moorean arguments. These are roughly arguments which rely on some method or faculty to prove its own reliability, such as the following:

Bootstrapping^11^

1. Know (Tank is full at t1) *(Reliable Process)*
2. Know (Gauge reads “F” at t1) *(Perception)*
3. Know (Gauge reads “F” at t1 & Tank is full at t1) *(Logical Inference)*
4. Know (Gauge reads accurately at t1) *(Logical Inference)*
5. Repeat
6. Know (Gauge is reliable) *(Induction)*

These don’t seem to be good arguments, in the sense that they do not seem to enable one to know their conclusions. One motivation for the sensitivity condition is that it can explain what goes wrong in cases of bootstrapping arguments, namely that their conclusions are insensitive. Melchior, however, aims to undermine this motivation by showing that although the conclusions of *some* bootstrapping arguments are insensitive, not *all* of them are.

Firstly he aims to look at the good case for sensitivity, where the conclusion of the argument is insensitive. He argues that this is the case when the step in the argument analogous to step (4) (above) is of the form ~ (Bp & ~ p), since beliefs of this form are insensitive. We have seen already that he is wrong to think this, so let us move swiftly over this point.

Next he aims to look at the bad case for sensitivity, where the conclusion of the bootstrapping argument is sensitive. He argues that this is the case when it involves beliefs of the form (Bp & p):

1. B(p1)
2. B(I believe that p1)
3. B(My belief that p1 is true)
4. Repeat for p2 … pn
5. B((My belief that p1 is true)…(My belief that pn is true))

Given what he has already said about beliefs of the form (Bp & p), he states that the conclusion of this argument is sensitive (so long as each of the beliefs in step 1 of the argument are). So the conclusion of this bootstrapping argument is sensitive and that is bad news for sensitivity.

But is this ‘bootstrapping’ argument really deserving of the name? I think not. The concrete example Melchior gives of this kind of argument has the following conclusion:

- (5) (My belief that COMPUTER is true), (My belief that DESK is true) …

Where COMPUTER, DESK, etc. are a series of mundane propositions taking note of the objects in front of him. The thing is that this just looks like something that he *does* know - this isn’t really ‘bootstrapping’ at all! Given (as he allows) that one can know of some belief one holds that it is true, there seems to be nothing wrong with the claim that one can know such a conclusion (which just conjoins several such pieces of knowledge).^12^

## Conclusion

There is no ‘the heterogeneity problem’ for sensitivity. Sensitivity does not treat relevantly similar higher-order beliefs differently (it only treats differently those that clearly embed a skeptical hypothesis - as it should). Likewise, the kind of ‘bootstrapping arguments’ that Melchior shows to have sensitive conclusions aren’t really ‘bootstrapping’ at all. (Sensitivity remains able to explain all cases of *actual* bootstrapping.) Whatever other problems the sensitivity condition may have, ‘the heterogeneity problem’ is not among them.

## Footnotes

1 Melchior (2015).

2 Nozick (1981).

3 Becker (2006), Salerno (2010).

4 B is a belief operator, p is a proposition, ~ stands for negation, and & for conjunction.

5 Again, this is in line with Becker and Salerno.

6 Vogel (1987, 2000, 2007, 2012); Sosa (1999, 2002) and Huemer (2001) endorse essentially the same argument.

7 Melchior (2015), p.489.

8 I also argue for this point in Wallbridge (2016a).

9 Cross (2007) makes a similar point about this case.

10 Could one insist that applying the same method necessarily involves basing one’s belief *on the exact same (subjective) evidence*? In short, no. This ends up being a wildly implausible interpretation of the sensitivity condition, on which no belief would ever be sensitive: take anything you believe, if that were false, *but* you formed a belief on the matter on the basis of evidence indiscriminable from your actual evidence (which was somehow still available), you would still believe it.

11 Melchior (2015), p.484.

12 He goes on to say that the same structure applies to an argument with the conclusion: *(5”) (COMPUTER and I am not a BIV deceived in falsely believing COMPUTER) and …* And it is especially jarring that this should be sensitive since it is so close to a direct claim that skeptical hypotheses are false. Melchior is right to think that the conclusion in this case is sensitive, and that this is an odd result. However, this problem is not due to bootstrapping: the oddity is already present in the premises of the argument (e.g. *COMPUTER and I am not a BIV*), it is odd that *these* are sensitive. Moreover, the problem does not stem from issues to do with higher-order knowledge: Nozick himself noted that his sensitivity condition makes simple conjunctions like *I am at home and not a BIV on Mars* sensitive. (Because all of the closest worlds in which this conjunction is false are ones in which one is simply not at home, and not ones in which one is in the misleading circumstance of being a BIV.) So although this may be a problem for sensitivity it is not a problem which has to do with bootstrapping or higher-order knowledge - additionally, see McBride (2014) for a revised sensitivity condition which avoids this problem. The delineation of belief forming methods proposed in Wallbridge (2016b) would also get around this problem.
